# Autophagy and inflammation an intricate affair in the management of obesity and metabolic disorders: evidence for novel pharmacological strategies?

**DOI:** 10.3389/fphar.2024.1407336

**Published:** 2024-06-04

**Authors:** Marzia Friuli, Christian Sepe, Elisabetta Panza, Cristina Travelli, Irene Paterniti, Adele Romano

**Affiliations:** ^1^ Department of Physiology and Pharmacology “V. Erspamer”, Sapienza University of Rome, Rome, Italy; ^2^ Department of Pharmacy, School of Medicine and Surgery, University of Naples Federico II, Naples, Italy; ^3^ Department of Pharmaceutical Sciences, University of Pavia, Pavia, Italy; ^4^ Department of Chemical, Biological, Pharmaceutical and Environmental Sciences, University of Messina, Messina, Italy

**Keywords:** autophagy, inflammation, obesity, metabolic diseases, therapeutic targets, diabetes, NAFLD, NASH

## Abstract

Unhealthy lifestyle habits including a sedentary life, the lack of physical activity, and wrong dietary habits are the major ones responsible for the constant increase of obesity and metabolic disorders prevalence worldwide; therefore, the scientific community pays significant attention to the pharmacotherapy of such diseases, beyond lifestyle interventions, the use of medical devices, and surgical approaches. The intricate interplay between autophagy and inflammation appears crucial to orchestrate fundamental aspects of cellular and organismal responses to challenging stimuli, including metabolic insults; hence, when these two processes are dysregulated (enhanced or suppressed) they produce pathologic effects. The present review summarizes the existing literature reporting the intricate affair between autophagy and inflammation in the context of metabolic disorders, including obesity, diabetes, and liver metabolic diseases (non-alcoholic fatty liver disease (NAFLD) and non-alcoholic steatohepatitis (NASH)). The evidence collected so far suggests that an alteration of autophagy might lead to maladaptive metabolic and inflammatory responses thus exacerbating the severity of the disease, and the most prominent conclusion underlies that autophagy might exert a protective function by contributing to balance inflammation. However, the complex nature of obesity and metabolic disorders might represent a limit of the studies; indeed, although many pharmacological treatments, producing positive metabolic effects, are also able to modulate autophagic flux and inflammation, it is not clear if the final beneficial effect might occur only by their mechanism of action, rather than because of additionally involved pathways. Finally, although future studies are needed, the observation that anti-obesity and antidiabetic drugs already on the market, including incretin mimetic agents, facilitate autophagy by dampening inflammation, strongly contributes to the idea that autophagy might represent a druggable system for the development of novel pharmacological tools that might represent an attractive strategy for the treatment of obesity and metabolic disorders.

## 1 Introduction

Macroautophagy (hereafter referred to as autophagy) is a conserved, self-degradative, and homeostatic process, mainly involved in the recycling and degradation of cellular components including damaged organelles, aggregated or misfolded proteins, lipids, and pathogens.

**FIGURE 1:**
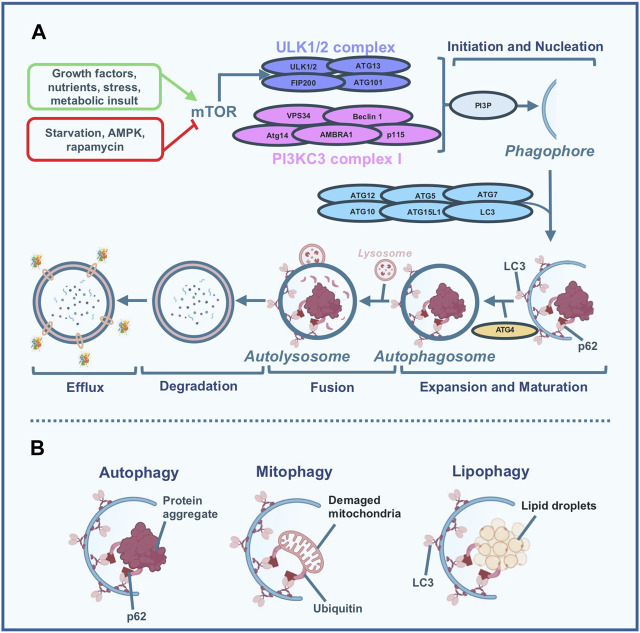
Simplified scheme of the autophagy process. **(A)** Different stimuli are capable of modulating autophagy flux. High nutrients, growth factors, stress, and metabolic insult inhibit the autophagic process by the activation of the mammalian target of rapamycin (mTOR) which in turn inhibits the uncoordinated 51-like kinase (ULK)-1/2 complex activity; nutrient deprivation, specific inhibitor of mTOR like rapamycin, and AMP-activated protein kinase (AMPK) induce autophagy. When autophagy is activated, the ULK1/2 and Beclin 1 - class III phosphoinositide 3-kinase (PI3KC3) complexes are required for the formation of the phagophore. Specifically, Beclin 1 forms a complex with PI3KC3 leading to the production of phosphatidylinositol-3-phosphate (PI3P) which represents a trigger of the subsequent nucleation phase. During nucleation, the phagophore (a membranous structure) is formed and different proteins, including Atg5, Atg12, Atg16L, LC3, ATG7, and ATG10 participate in the expansion and maturation of the phagophore in the autophagosome. Particularly, Atg4 is involved in the lipidation of the LC3, which is essential for the formation of the autophagosome. During the subsequent fusion step, the autophagosome, which contains the material to be degraded (either lipids, damaged mitochondria, damaged organelles, aggregated and misfolded proteins, or pathogens) conjugates with the lysosome to form the autolysosome. Once this step is completed, the degradation phase occurs by exposing the material enclosed in the autolysosome to the hydrolytic enzymes present in the lysosomes. Finally, in the last step, the efflux phase, the degraded material is released by the lysosome and the phagosome membranes. **(B)** Macroautophagy (usually named autophagy) is a multi-step conserved, self-degradative, and homeostatic process, mainly involved in the recycling and degradation of cellular components: we can refer to selective types of autophagy including mitophagy (degradation of damaged mitochondria) and lipophagy (degradation of lipid droplets).

When autophagy is activated a double-membraned vesicle known as the autophagosome, engulfs these cellular components and subsequently fuses with lysosomes to complete the degradation process, by involving the so-called autophagy-related proteins (ATGs), the mammalian target of rapamycin (mTOR), and the AMP-activated protein kinase (AMPK) (for review see (Zhang et al., 2018)). Figure 1 panel A depicts a simplified scheme of the autophagy process.

Autophagy is commonly recognized as a defense mechanism able to promote either cell survival or cell death by adapting its machinery to different stimuli (Sadeghi et al., 2023) including growth factors deficiencies, oxidative alterations, and metabolic changes. It is well known that autophagy is sensitive to nutrient status, and fluctuation in lipid level might serve as a physiological sensor for its regulation. Accumulating evidence suggests that autophagy is closely related to the control of energy homeostasis exerted by the hypothalamus, the brain area mainly involved in monitoring changes in the body’s energy state, by sensing alterations of key metabolic hormones and nutrients (Meng and Cai, 2011). Moreover, it is important to report that defective hypothalamic autophagy can employ changes in inflammatory pathways to exacerbate the development of obesity and metabolic disorders (Meng and Cai, 2011).

Indeed, it has been demonstrated that defects in autophagy homeostasis (an enhancement or a suppression) facilitate the onset and the development of metabolic disorders, including obesity, diabetes, and metabolic liver diseases (Kim and Lee, 2014; Zhang et al., 2018; Raza et al., 2023).

Interestingly, autophagy substrates can be preferentially sequestrated and degraded by selective types of autophagy, including lipophagy and mitophagy (Figure 1 panel B), (Zhang et al., 2018). While lipophagy is defined as the selective autophagic degradation of lipid droplets (LDs), mitophagy is a fundamental quality-control mechanism that specifically removes damaged mitochondria to maintain a functional mitochondrial network. Ectopic accumulation of fat, muscle insulin resistance, and β-cell dysfunction, the three main hallmarks occurring during obesity and type 2 diabetes mellitus (T2DM) mostly depend on the metabolic overload of mitochondria. During lipophagy triglycerides (TGs) and cholesterol are engulfed by the autophagosome and transported to lysosomes for their degradation to ultimately originate free fatty acids, which in turn, constitute fuel for mitochondrial β-oxidation. Therefore, lipophagy regulates not only intracellular lipid stores and cellular free lipids but furthermore governs cellular energy homeostasis by supporting the production of ATP. Importantly lipophagy is a selective mechanism since the quantity of lipids metabolized changes based on the extracellular source of nutrients; therefore, during an excess of nutrients (like what happens in obesity) impaired lipophagy can lead to an excess of ectopic lipid accumulation responsible for organ dysfunction and finally for cell death (for review see (Liu and Czaja, 2013)). In such conditions the β-oxidation of free fatty acid results insufficient, thus leading to the accumulation of toxic lipid intermediates which produce radical species responsible for oxidative stress associated with mitochondrial damage. The elimination of dysfunctional mitochondria via mitophagy is a process of specific importance during altered metabolic status since it allows the elimination of the vicious cycle of oxidative stress and thus counteracts the development of pathogenic processes (for review see (Sarparanta et al., 2016)).

Hence, besides autophagy, the cellular response to stressors recruits other pathways, among which the most important is inflammation. In the classic literature, inflammation is defined as the primary response of the body to address injuries. This short-term adaptive response is essential for tissue repair and involves the integration of several complex signals in distinct cells and organs. However, the extended consequences of persistent or prolonged inflammation are frequently detrimental and can alter normal physiological processes by contributing to the development of pathological chronic conditions (Hotamisligil, 2006).

A variety of studies suggest that an alteration of autophagy pathways represents an important issue in several inflammatory conditions including metabolic-related diseases (Kocak et al., 2022) and a reciprocal relationship between these two processes is crucial to orchestrate fundamental aspects of cellular and organismal responses to dangerous stimuli (Gukovsky et al., 2013; Wang et al., 2013). Indeed, several components partaking in the inflammatory cascade, including Toll-like receptors (TLRs), NOD-like receptors (NLRs), and pro-inflammatory cytokines, show the capability to activate autophagy. Conversely, also autophagy plays a regulatory role in inflammation by influencing the secretion of pro-inflammatory cytokines, the activation or inhibition of inflammasomes, and by shaping the composition of immune cells within tissues (Pang et al., 2022).

The intricate interplay between autophagy and inflammation appears crucial to orchestrate overall cellular homeostasis and efficiently respond to several challenges (Qian et al., 2017).

The present review summarizes the existing literature reporting the intricate affair between autophagy and inflammation in the context of metabolic diseases, including obesity, diabetes, and liver metabolic diseases such as non-alcoholic fatty liver disease (NAFLD) and non-alcoholic steatohepatitis (NASH). The understanding of the interconnected nature of these two processes in such a context might help to identify novel pharmacological tools that might represent an attractive strategy for the treatment of obesity and other metabolic diseases.

## 2 Obesity

Although the assessment of autophagy in obesity is challenging and the results reported in the literature are often controversial, a consistent number of studies report that altered autophagy has been observed in obese patients and animal models of obesity (Zhang et al., 2018). One of the most sustained hypotheses is that inhibition of autophagy enhances proinflammatory gene expression, thus suggesting that autophagy may serve to dampen inflammation and thereby limit excessive inflammatory response during obesity (Jansen et al., 2012). Most of the studies suggest that the brain’s hypothalamic area and the adipose tissue are the central stations crucial for the autophagy processes in obesity.

### 2.1 Hypothalamic autophagy

Accumulating evidence suggests that autophagy is closely related to the control of energy homeostasis exerted by the hypothalamus (Romano et al., 2020a) and impaired autophagy is observed in the hypothalamic arcuate nucleus when mice are challenged with a high-fat diet (HFD) (Meng and Cai, 2011). Moreover, hypothalamic knockdown of *Atg7* gene (which plays a key role in the autophagic response), leads to hyperphagia associated with an increase in body weight, a decrease of energy expenditure, and the activation of the proinflammatory IkappaB kinase beta (IKKβ) pathway (Meng and Cai, 2011). Moreover, by using brain-specific IKKβ knockout mice, it has been demonstrated that altered hypothalamic autophagy is reversed by the inhibition of IKKβ pathway in the brain, thus further suggesting that inflammation influences the autophagy process (Meng and Cai, 2011).

The hypothalamus contains neurons that release specific neuropeptides that generate behavioral responses including the initiation or cessation of feeding; a variety of elegant studies suggest that hypothalamic autophagy has a great influence on both metabolism and eating depending on the types of peptidergic neurons affected (Kaushik et al., 2011; Parker and Bloom, 2012). For instance, it has been demonstrated that the activation of autophagy in cultured hypothalamic neurons in a starved state induces a mobilization of lipids which generate free fatty acids that in turn increase the level of the hypothalamic orexigenic peptide agouti-related protein (AgRP) that stimulate eating (Kaushik et al., 2011). On the other hand, it has been shown that a specific inhibition of autophagy in AgRP neurons of mice induces a decrease in fasting-increasing AgRP levels and a reduction of food intake (Kaushik et al., 2011). Additionally, mice with AgRP neuron-specific deletion show a decrease in body weight and adipose mass which is associated with an increase in the levels of both the anorexigenic pro-peptide proopiomelanocortin and an increase of its cleavage product, the α-melanocyte stimulating hormone (Kaushik et al., 2011). On the same line, also the hypothalamic neuropeptide Y (NPY) neurons seem to be under the control of autophagic processes in the presence of a metabolic insult (Reginato et al., 2020). In this context, Reginato and collaborators (Reginato et al., 2020) suggested that neurons sense fatty acid content and in turn regulate autophagy. However, the toxic effects of saturated fatty acids infusion are not perceived during acute exposure, when hypothalamic autophagy remains functional and serves as a protective mechanism to support neuronal homeostasis, rather than during chronic exposure. In this latter condition, the authors suggest that intracerebroventricular (i.c.v.) infusion of the saturated lipid acid palmitate (PA) alters the gene expression of the orexigenic peptide NPY which is responsible for an increase in feeding and ultimately leads to obesity (Reginato et al., 2020). Moreover, i.c.v. PA exposure increases Atg7 and LC3B positive cells in NPY neurons and this effect is associated with a decrease in hypothalamic levels of pS6, a downstream marker of mTOR complex 1, thus suggesting an induction of autophagy (Reginato et al., 2020). Interestingly such an effect was associated with a dysregulation in the mRNA levels of hypothalamic pro-inflammatory cytokines and endoplasmic reticulum (ER) stress markers.

### 2.2 Adipose tissue autophagy regulation

Despite the role of autophagy in the control of the hypothalamic regulation of energy metabolism, several studies have been conducted in the adipose tissue, and a great number of them have pointed out that autophagy also participates in adipogenesis (Singh et al., 2009b; Zhang et al., 2009). The contribution of autophagy to adipocyte differentiation has been demonstrated in more than one study and different experimental contexts; indeed *in vitro* knockdown of *Atg7* gene in mouse adipocytes leads to an inhibition of autophagic function, to a decrease of TGs content and a reduction of the expression of adipocyte differentiation markers (Singh et al., 2009b). On the same line, adipose-specific deletion of *Atg7* in mice leads to a lean phenotype which is associated with a reduction in white adipose tissue (WAT) and a conversion from a white adipocyte phenotype to a brown one. The increase of the brown adipose tissue (BAT) results in a catabolic effect deriving from an improved insulin sensitivity and an increase of fatty acid β-oxidation (Singh et al., 2009b). In keeping with this latter study, other authors also showed that *Atg7* knockout mice are resistant to the development of obesity-induced by HFD exposure and show an increase in insulin sensitivity and a decrease in leptin plasma concentration (Zhang et al., 2009).

The expansion of the adipose tissue during obesity is associated with an important inflammatory state occurring in adipocytes which contributes to the establishment of a chronic low-grade systemic inflammation typical of obesity. In this regard, Jansen et al. (2012) have demonstrated that the inhibition of autophagy increases the gene expression and secretion of proinflammatory cytokines, including interleukin (IL)-1β, IL-6, and IL-8 in the adipocytes of obese people and in the adipose tissue of obese mice, thus suggesting that autophagy may dampen excessive inflammation in adipose tissue during obesity. Moreover, in a very recent study (Hosseini et al., 2024) the authors investigated the role of autophagy in eosinophils, immune cells strongly involved in adipose tissue homeostasis. They used a genetic mouse model lacking *Atg5* specifically within the eosinophils and they observed that the absence of *Atg5* was associated with an increase in body weight and alterations of the adipose tissue cellular architecture. Moreover, by using an *in vitro* approach, they also demonstrated a pro-inflammatory shift in macrophages co-cultured with *Atg5*-knockout eosinophils, shedding new light on the role of autophagic process in eosinophils and its impact on adipose tissue homeostasis and adipose tissue inflammation.

Among the factors influencing inflammation associated with obesity, the NLRP3 inflammasome, one of the most well-studied and characterized inflammasomes, has emerged as a potential central hub that assembles in response to cellular perturbations. Its activation stimulates caspase-1, which in turn promotes the maturation and the release of inflammatory cytokines including the IL-1β and IL-18. These cytokines participate in the development of a systemic low-grade inflammation typical of the obese state. Indeed, increased levels of NLRP3 inflammasome were observed in obesity (Zhu and Liu, 2022). In this regard, in a recent study, Ko and colleagues (Ko et al., 2021) demonstrated that the deletion of *pink1*—a gene involved in selective mitochondrial autophagy (mitophagy)—either globally or specifically in brown adipocytes, resulted in the dysfunction of BAT and a marked increase in weight gain in mice fed with HFD compared to the control group (wild type fed with HFD). They also demonstrated that impaired clearance of damaged mitochondria produced the induction of NLRP3 inflammasome and consequently the dysfunctional state of the BAT. Interestingly, *nlrp3* deletion in *pink1* knockout mice reversed BAT dysfunction, suggesting that targeting NLRP3 has the potential for therapeutic benefit in this context as a specific therapeutic target.

### 2.3 Summary

**FIGURE 2:**
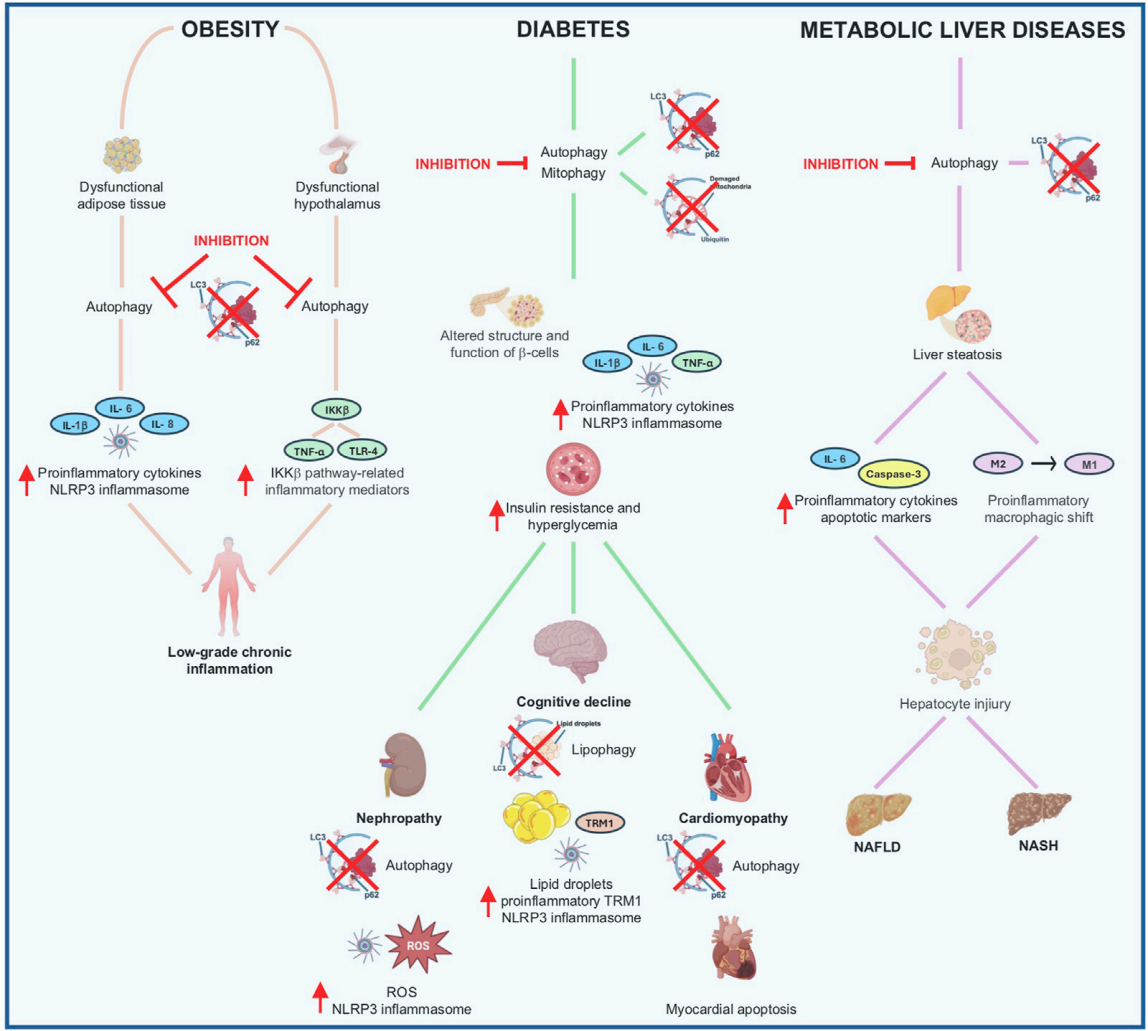
Autophagy and inflammation signaling mechanism in metabolic disorders Obesity: hypothalamic dysfunctional autophagy is associated with increased specific proinflammatory pathways including IKKβ signaling and its related inflammatory mediators. Inhibition of autophagy in adipose tissue results in adipose tissue dysfunction associated with increased inflammatory mediators including proinflammatory cytokines and NLRP3 inflammasome. These alterations are responsible for the establishment of a chronic low-grade systemic inflammation typical of obesity. Diabetes: inhibition of autophagy and mitophagy is associated with structural and functional alterations of pancreatic β-cells, and inflammatory changes (activation of proinflammatory cytokines and NLRP3 inflammasome) leading to hyperglycemia and insulin resistance. The sustained hyperglycemia affects cells and tissues in the entire body leading to an inhibition of autophagy associated with severe complications, including nephropathy (increased levels of ROS and NLRP3 inflammasome), cardiomyopathy (enhancement of myocardial apoptosis) and cognitive decline (impaired lipophagy in microglia associated to the activation of NLRP3 inflammasome). Metabolic liver diseases: inhibition of autophagy is associated with increased TGs and cholesterol liver content which leads to excessive accumulation of dietary lipids in the liver, also defined as steatosis. Persistent or prolonged steatosis induces inflammatory infiltration (e.g., proinflammatory IL-6 cytokine) and apoptotic markers (e.g., Caspase-3), and increases proinflammatory liver macrophage polarization from M2 to M1 phenotype. These proinflammatory events produce hepatocyte injuries resulting in NAFLD and NASH, which can ultimately lead to advanced fibrosis, hepatic cirrhosis, and liver failure.

Most of the studies collected in this paragraph are focused on hypothalamic and adipose tissue level, two stations critical for the control of energy homeostasis and eating behavior. The evidence collected at the hypothalamic level suggests that i) lipids can alter hypothalamic autophagic pathways by recruiting different orexigenic and anorexigenic neuropeptides that govern energy homeostasis and eating behavior, ii) inflammation might work in concert with autophagy as a mechanism to support the control of energy homeostasis exerted by the hypothalamus. On the other side, the overall conclusion derived from the studies conducted on the adipose tissue supports the idea that autophagy may dampen excessive inflammation in adipose tissue during obesity; autophagy seems to play a crucial role in adipocyte differentiation/development and in the balance between WAT and BAT. This information sheds new light on potential mechanisms associated with autophagy and inflammatory processes in the adipose tissue. Finally, although the studies reported in this paragraph are often controversial making the assessment of autophagy in obesity very challenging, most of them converge on the hypothesis that autophagy may operate to reduce inflammation and thus limit the chronic inflammatory state occurring in obesity. The evidence collected in this paragraph is summarized in Figure 2.

## 3 Diabetes

Diabetes represents one of the most widespread metabolic diseases worldwide and hyperglycemia is the common outcome of the two main forms of this disorder: type 1 diabetes mellitus, characterized by autoimmune destruction of pancreatic β-cells, and (T2DM), the most common, characterized by insulin resistance and a poor efficacy of insulin to control glucose homeostasis (Eizirik et al., 2020). T2DM is often associated with an exacerbation of the obese condition leading to the so-called metabolic syndrome. In this context the adipose tissue seems to be crucially involved in the production of inflammatory biomarkers, resulting from a crosstalk between immune cells, adipocytes, and macrophages that permeate it. Inflammatory response contributes to the development of T2DM by inducing insulin resistance (Lontchi-Yimagou et al., 2013). Interestingly, epidemiologic studies have suggested an association between inflammation and the occurrence of T2DM and its complications (Ruze et al., 2023). It is known that autophagy crucially contributes to the structural and functional maintenance of pancreatic islets (Yasasilka and Lee, 2024). In this context, Sidarala and collaborators suggest that mitophagy supports β cell survival and prevents the development of diabetes by dampening inflammation and that the pharmacological targeting of such pathway might potentially result in the prevention of β cell dysfunction in diabetes (Sidarala et al., 2020).

Interestingly in a state of high glucose concentration, autophagy is inhibited, and islet functions are impaired, leading to insulin resistance (Gonzalez et al., 2011). In this regard, studies have suggested that the regulation of autophagy might result in a positive outcome for the treatment of T2DM and its related pathological alterations (for review see (Zhao et al., 2023)). Specifically, in mice with a selective deletion of Atg7 in β-cells, structural alterations and reduced insulin secretion have been observed. Morphologic analysis showed an increase of ubiquitinated protein aggregates which colocalize with p62, considered a “sensor” of autophagy that drives and delivers protein aggregates to the autophagosomes (Jung et al., 2008).

In a recent paper, Park and collaborators (Park et al., 2020) by using a genetic model of mild insulin resistance, the ob/ob mice, observed an increase of hypothalamic ER stress, starting at postnatal day 10, before the development of obesity. Interestingly cells exposed to ER stress often activate autophagy and, on this line, the authors found that *in vitro* induction of ER stress by using tunicamycin, stimulates in the hypothalamus the autophagy-related genes *Atg5*, *Atg7*, *Atg12*, and *p62*. Interestingly the pharmacological treatment by relieving ER stress, ameliorates the glucose homeostasis, body weight, and food intake, thus highlighting the existence of an ER stress-autophagy pathway able to influence hypothalamic circuits and metabolism. Therefore, Lim and collaborators (Lim et al., 2014) showed that mice with global haploinsufficiency of the gene *Atg7* (Atg7 (+/−) mice) develop diabetes when crossed with the ob/ob mice. Particularly, Atg7 (+/−)-ob/ob mice display an exacerbation of insulin resistance compared to ob/ob mice which is associated with an increase of liver lipid content and inflammatory changes (analyzed into the liver, WAT, and muscle) with activation of inflammasome. The pharmacological enhancement of autophagy flux by using imatinib or trehalose (two compounds able to enhance autophagy (Park and Lee, 2022)) results in a therapeutic effect, by improving metabolic parameters. This study strongly suggests that inhibition of systemic autophagy might impair the adaptive response to metabolic stress and might represent a causal factor in the shift from obesity to diabetes.

### 3.1 Autophagy regulation in diabetic complications

It is important to mention that the sustained hyperglycemia occurring during diabetes affects several cells and tissues in the entire body and induces an oxidative stress response leading to severe complications, including nephropathy, cardiomyopathy, and cognitive decline (Zhao et al., 2023). It is well known that neuroinflammation induced by microglial activation results in neurological disorders which are prominent aspects of cognitive impairments associated with diabetes. Studies reveal that microglial lipophagy, an important part of autophagy that participates in lipid homeostasis and inflammation could be an Achilles’s heel in diabetes-associated cognitive impairment (Li et al., 2023). An increase in glucose levels can impair lipophagy and lead to an accumulation of LDs in the microglia of different animal models of diabetes. Interestingly LDs accumulation in the microglia colocalized with the inflammatory amplifier, the triggering receptor expressed on myeloid cells (TREM)1, which in turn promotes neuroinflammation and exacerbates hyperglycemia-inducing lipophagy, and behavioral cognitive impairment via the inflammasome NLRP3. The pharmacological blockade of TREM1 can revert such conditions by reducing inflammatory damage, and consequently improving cognitive functions. These results suggest that targeting lipophagy-induced inflammation in diabetes preclinical models might represent a therapeutic strategy for delaying diabetes-associated cognitive decline (Li et al., 2023).

As already reported before in this paragraph, diabetes is often associated with cardiomyopathy; in this regard, it has been suggested that in a pharmacological model of diabetes obtained by treating the animals with streptozotocin, mice show an enhancement of myocardial apoptosis associated with an inhibition of autophagy in the heart. Such effect is completely reversed by the knockdown of the transient receptor potential melastatin 2 gene which modulates autophagy processes (Hu and Lin, 2024). Also, the kidney is an organ that might undergo pathological changes during a condition of hyperglycemia, and a very complex and recent study evidenced that inflammation, the increase of oxidative stress, and altered autophagy contribute to the development of diabetic kidney disease (Jianbing et al., 2022). The result of the study suggests that the pharmacological treatment of human renal glomerular endothelial cells with rapamycin, an autophagy inducer, can dampen the levels of reactive oxygen species (ROS) and inflammation induced by high concentrations of glucose, while autophagy inhibitors show the opposite function. These results suggest autophagy as a new druggable system for the treatment of diabetic kidney disease (Jianbing et al., 2022).

### 3.2 Summary

The evidence collected in the present paragraph suggests that autophagy not only plays a pivotal role in maintaining the physiologic pancreatic islet architecture but is a crucial controlling signaling pathway for T2DM and its complications since it regulates glucose metabolism and insulin secretion. In particular, autophagy promotes β-cell survival by allowing adaptive responses to avoid or alleviate the negative effects of mitochondrial dysfunction, ER stress, and oxidative stress occurring in T2DM. The evidence collected in this paragraph is summarized in Figure 2.

## 4 Metabolic liver diseases

As an evolutionarily preserved process, autophagy can be found in all the eukaryotic cells including the hepatocytes, where, as a catabolic process, it contributes to the preservation of liver tissue homeostasis in both parenchymal (hepatocytes) and non-parenchymal (Kupffer cells, hepatic stellate cells, and sinusoidal endothelial cells) hepatic cells (Raza et al., 2023). The understanding of cell type-specific autophagy in the liver is important to develop novel focused pharmacological targets for the treatment of liver diseases. As already mentioned before in the text, autophagy participates in lipid metabolism, known as lipophagy, and interestingly mice lacking *Atg7* gene or pharmacologically treated with an autophagy inhibitor show an increase in liver TGs and cholesterol (reviewed in (Ward et al., 2016)). On the same line, hepatocyte cells pharmacologically treated with an autophagy inhibitor or knockdown for the autophagy gene *Atg5*, show increased TGs accumulation (Singh et al., 2009a). The excessive accumulation of dietary lipids in the liver, also defined as steatosis, is responsible for disrupted hepatic lipid homeostasis with an accumulation of hepatic TGs, a typical feature of NAFLD (Giudetti et al., 2021) more recently also named metabolic dysfunction-associated steatotic liver disease. Persistent or prolonged steatosis can induce inflammatory infiltrate and hepatocyte injuries (e.g., focal necrosis and fibrosis), resulting in NASH, which can ultimately lead to advanced fibrosis, hepatic cirrhosis, and liver failure (Kořínková et al., 2020). Therefore, the regulation of excessive lipid accumulation in the hepatic tissue is crucial in the prevention or treatment of liver diseases including NAFLD and NASH. To support such a hypothesis, it has been observed an impaired autophagic flux both in the liver of a murine model of NASH and in liver biopsy of patients affected by NASH, as well as in lipid-overloaded human hepatocytes (González-Rodríguez et al., 2014). Moreover, Hammoutene et al. (Hammoutene et al., 2020) demonstrated that defective autophagy (deficiency of *Atg5*) in hepatic endothelial cells is associated with the promotion of liver inflammation (assessed by upregulation of cytokines including IL-6 and an increase of apoptotic markers) and fibrosis both in the early and the advanced stages of NASH. On the same line, the lack of *Atg5* specifically within liver macrophages (also known as Kupffer cells) of mice exposed to HFD and treated with low-dose LPS induces a pro-inflammatory phenotype resulting in an increase of proinflammatory M1 and a decrease of anti-inflammatory M2 polarization (Liu et al., 2015). These latter studies suggest a pivotal role of *Atg5* gene in the management of hepatic inflammatory state leading to the progression of liver injury.

It is well known that liver injury is responsible for hepatocyte death, leading to liver failure, hepatic fibrosis, and in the worst cases hepatocellular carcinoma; in some liver diseases including nonalcoholic and alcoholic steatohepatitis, hepatocyte death happens by a variety of mechanisms including apoptosis, necrosis, and necroptosis (Luedde et al., 2014).

NAFLD is also associated with the activity of nitric oxide synthase (NOS), and inducible NOS (INOS), two endogenous molecules often associated with liver pathological consequences, among which the promotion of lipid peroxidation and mitochondrial damage, the inhibition of hepatocyte protein synthesis and glucose homeostasis, all events which accelerate hepatocyte death. The evidence collected in a mouse model of NAFLD suggests that the liver expression of INOS is increased in macrophages and conversely the INOS knockdown decreases the macrophages number; this latter effect is associated with an improvement of autophagy observed by an increase of the autophagy-related molecules LC3 in macrophages which contribute to the maintenance of intracellular liver homeostasis during NAFLD (Jin et al., 2023).

Finally, the prevalence of NAFLD and the lack of an adequate therapy urge to also seek the identification of biomarkers of the pathology for a timelier diagnosis leading to a more efficacious pharmacological treatment. On this line, it is important to mention a very recent study published in January 2024 which comprises both preclinical and clinical evidence (Wu et al., 2024). The authors identify the upregulation of annexin 2 (ANXA2, a member of a super-family of calcium-dependent membrane binding proteins participating in a variety of fundamental biological processes, including cell survival and apoptosis) as a biomarker of NAFLD progression since its expression was increased in both the serum and the liver of patients and mice with NAFLD. By exploring the biological underpinning of such observation, the authors show that an increase of ANXA2 is stimulated by the inflammatory TLR4 cascade and negatively impacts the autophagic AMPK/mTOR pathway (Wu et al., 2024). Taken together the result of this last study underly that liver autophagy might represent the missing link between liver inflammation and the progression of liver chronic diseases.

### 4.1 Summary

Given the complex cellular composition of the liver and the related different responses to pathophysiological stimuli, further understanding of the pathways regulating hepatocyte damage is needed to unravel novel pharmacological treatments in such a context. However, the results reported in this paragraph suggest that the concerted participation of autophagy and inflammation seems to be fundamental in the management of lipid accumulation in the hepatic tissue and, consequently, of the hepatic inflammatory state leading to hepatocyte death. The evidence collected in this paragraph is summarized in Figure 2.

## 5 Therapeutic potential of the pharmacological modulation of autophagy

Unhealthy lifestyle habits including a sedentary life, the lack of physical activity, and wrong dietary habits are the major ones responsible for the constant increase of metabolic disorders prevalence worldwide, and the scientific community pays significant attention to the pharmacotherapy of the treatment of such metabolic diseases (Domingo et al., 2024). In this regard, overweight and obesity represent, in most cases, the main drivers of other metabolic disorders (Godoy-Matos et al., 2020) and most of the pharmacological treatments available for obesity, T2DM, and metabolic liver injury, including NAFLD and NASH, have a mutual effect on the others, although to date NAFLD and NASH do not have a recognized selective pharmacological treatment representing an unmet medical need (Kořínková et al., 2020; Ruze et al., 2023). Indeed, approved anti-obesity/antidiabetic drugs have also been evaluated in clinical trials for the treatment of NAFLD patients; these medications include glucagon-like peptide (GLP)-1 receptor agonists and the orlistat, a gastrointestinal lipase inhibitor. Interestingly the GLP-1 receptor (GLP1R) agonists liraglutide and semaglutide as well as the orlistat have shown beneficial effects on the two hits typical of NAFLD, the hepatic steatosis and the inflammatory process (for review see (Polyzos et al., 2022)). For instance, GLP1R agonists and orlistat reduce the production of proinflammatory adipokines, chemokines, and cytokines as well as their circulating levels (Domingo et al., 2024). Moreover, by unraveling the molecular mechanisms behind the antidiabetic and anti-obesity therapeutic effect of both GLP-1 agonists and orlistat, the researchers find that these medications can affect the autophagy flux, thus supporting that the evidence collected in the present review might represent a concrete perspective for future pharmacological interventions (Rowlands et al., 2018; Park and Lee, 2022; Domingo et al., 2024). Specifically, it has been shown (Fan et al., 2018) that, the antidiabetic effect of liraglutide is associated with an improvement of pancreatic defective autophagy obtained by the upregulation of ATG5 and LC3 expression in a mouse model of T2DM. In support of such a hypothesis, in another study (He et al., 2016) liraglutide was shown to improve hepatic steatosis associated with NAFLD, by inducing autophagy flux, and such effect was also associated with a decrease in body weight and decreased accumulation of TGs and cholesterol. This result is also supported by an *in vitro* experiment (performed in hepatocytes in which steatosis was induced by free fatty acids exposure) suggesting that liraglutide has a protective effect on hepatic steatosis which is mediated by the activation of autophagy flux particularly throughout the AMPK/mTOR pathway (Liao et al., 2024). The same conclusion has been achieved in another set of experiments where the authors demonstrated that liraglutide alleviates NAFLD by restoring autophagy via improving lysosomal function (Fang et al., 2020). Also, sitagliptin, a dipeptidyl-peptidase 4 inhibitor, has been reported to exert more biological properties beyond its antidiabetic effect; in particular, it has been shown to ameliorate the development of liver steatosis by inhibiting inflammatory responses and by activating autophagy via AMPK/mTOR pathway (Zheng et al., 2018). Although experiences in a different context, also the incretin-mimetic semaglutide and orlistat have been shown to affect the autophagy flux (Peng et al., 2018; Chang et al., 2020; Liu et al., 2022), thus further corroborating the idea that the therapeutic effect of such medicaments might be at least in part due to the modulation of autophagy.

Interestingly also the anorexigenic compound, oleoylethanolamide (OEA), a high-affinity agonist of the peroxisome proliferator-activated receptor alpha (PPAR-alpha), highly studied in the last 2 decades for its beneficial effect on food intake, lipid metabolism, and body weight (Romano et al., 2015; 2017; 2020b) has been suggested, although indirectly, to affect autophagy. Therefore, it has been demonstrated that OEA possesses a beneficial effect in vascular calcification induced by metabolic dysfunctions via inhibiting ferroptosis, a specific type of cell death dependent on autophagy; such effect was strictly dependent on the activation of PPAR-alpha (Chen et al., 2023). This latter result acquires considerable importance if we consider that the PPAR-alpha agonists i) GW7647 reverse feeding-induced autophagy suppression (Lee et al., 2014) and ii) fenofibrate, ameliorates HFD-induced kidney injury in diabetic kidney disease via autophagy activation (Sohn et al., 2017), thus supporting the role of PPAR-alpha, already known to regulate fatty acid metabolism, in activating autophagy. Also, the antidiabetic inhibitors of the sodium-glucose cotransporter 2 (SGLT2) have been demonstrated to augment autophagy in preclinical experiments performed in animals and cell cultures. In particular, SGLT2 inhibitors produce beneficial effects on the progression of both cardiomyopathy and nephropathy associated with diabetes by enhancing the autophagic flux in different tissues and organelles including the mitochondria. The enhancement of autophagy induced by SGLT2 inhibitors is accompanied by a reduction in oxidative stress, restoration of functional mitochondria, and a decrease in proinflammatory mediators thus contributing to the maintenance of physiological organ morphology and functions [for review see (Packer, 2022)]. Additionally, the research is also concentrated on the study of direct (Park and Lee, 2022) autophagy enhancers, including berberine, imatinib, and rapamycin in the potential treatment of metabolic diseases. In this regard, Panigrahi and Mohanty (Panigrahi and Mohanty, 2023) recently reported the results obtained from a randomized controlled trial (CTRI/2021/12/038751) on the effect of berberine in prediabetic patients. Particularly, the author showed that after 12 weeks of daily oral berberine (HIMABERB^®^) treatment, the glycemic markers were significantly improved thus supporting the potentiality of a direct enhancement of autophagy in the delaying of diabetes progression.

Although the present review is focused on the pharmacological modulation of autophagy to dampen inflammation in metabolic diseases, it is worth mentioning that also non-pharmacological approaches including diet management, fasting, and exercise have been studied for their ability to affect autophagy in such a context. For instance, it has been demonstrated that caloric restriction and physical activity serve as potent mechanisms for upregulating autophagy (van Niekerk et al., 2018), resulting in being dampened by excessive nutrition and a sedentary lifestyle. In particular, fasting can enhance mitophagy, by facilitating the removal of damaged mitochondria, and consequently by reducing ROS production (van Niekerk et al., 2018). Moreover, it has also been shown that exercise triggers autophagy in multiple organs that play a key role in metabolic regulation, including muscle, liver, pancreas, and adipose tissue (He et al., 2012), thus highlighting the importance of physical activity in a daily routine to improve health. Further research, also in nutrition, has studied different nutrient approaches as activators of autophagy. For instance, a link between the ketogenic diet and autophagy activation has been demonstrated in a preclinical study where the mice were fed a ketogenic diet for 4 weeks, which induced an enhancement of autophagy in hepatocytes (for review see (Kocot and Wróblewska, 2022)). Finally, several reports elucidate autophagy-related signal pathways of functional foods, including some bioactive components such as resveratrol, epigallocatechin-3-gallate, and curcumin (for review see (Xie et al., 2019)).

Further discussion on such non-pharmacological interventions is out of the aim of the present review although they seem promising in supporting traditional pharmacological treatments for diseases related to dysregulated autophagy including obesity and metabolic disorders.

## 6 Conclusions and future perspectives

The studies collected in the present review provide evidence of the sophisticated affair between inflammation and autophagy in modulating biological events regulating energy homeostasis and metabolism. The most prominent conclusion mainly coming from the preclinical studies (summarized in Table 1) is that autophagy exerts a protective function by contributing to balance inflammation occurring in obesity and metabolic diseases; on the contrary, an altered autophagy might lead to maladaptive metabolic and inflammatory responses thus exacerbating the severity of the disease.

**TABLE 1:** Autophagy and inflammation in obesity and metabolic disorders: a scheme of the most prominent results reported in the manuscript.

Subjects	Autophagy/Inflammaton	Other effects	References
Obesity			
*Atg7*- KO mice	↓ autophagy; ↑ IKKβ mediated inflammation	↑ body weight and ↓ energy expenditure	Meng and Cai (2011)
Lipid i.c.v. infused mice	↑ Atg7 and LC3B positive cells in NPY neurons; ↑ pro-inflammatory cytokines genes	↑ food intake	Reginato et al. (2020)
Obese mice and humans	↓ autophagy; ↑ proinflammatory cytokines	↑ body weight and ↓ insulin sensitivity	Jansen et al. (2012)
Eosinophils *Atg5*-KO mice	↓ autophagy; pro-inflammatory shift of macrophages	↑ body weight; altered adipose tissue architecture	Hosseini et al. (2024)
*pink1*-KO mice fed with HFD	↓ mitophagy; induction of NLRP3 inflammasome	↑ body-weight gain; alteration of BAT structure and function	Ko et al. (2021)
Cellular model of starvation in hypothalamic neurons	↑ autophagy	↑ endogenous free fatty acids; downregulation of the orexigenic AgRP effect in response to starvation	Kaushik et al. (2011)
Diabetes			
Diabetes animal models	↓ lipophagy; ↑neuroinflammation	↑ glucose levels and ↑cognitive impairment	Li et al. (2023)
Atg7 (+/−)-ob/ob mice	↓ autophagy; ↑ inflammasome	Insulin resistance and ↑ liver lipid content	Lim et al. (2014)
Beta-cells *Atg7*-KO mice	↓ autophagy; ↑ beta-cells apoptosis	↓ insulin secretion and alteration of beta-cells structure and mass	Jung et al. (2008)
Streptozotocin-induced diabetes mice model	↓ autophagy; ↑ myocardial apoptosis	↑ glucose levels	Hu and Lin (2024)
Human glomerular endothelial cells treated with rapamycin	↑autophagy; ↓ ROS and inflammation	↓ glucose levels	Jianbing et al. (2022)
Metabolic liver diseases			
Murine model of NASH, patients affected by NASH	↓ autophagic flux	↑ body weight	González-Rodríguez et al. (2014)
		↓ HDL-cholesterol levels	
Hepatic endothelial cells *Atg5*-KO mice	↓ autophagy; ↑ inflammation	↑ liver fibrosis	Hammoutene et al. (2020)
Murine peritoneal macrophages INOS-KO stimulated with lipid	↑autophagy; ↓ proinflammatory cytokines	Regulation of lipid homeostasis	Jin et al. (2023)
Mice model of NAFLD with ANXA2-KO	↑ autophagic flux	↓ body weight, liver volume and hepatic lipid accumulation	Wu et al. (2024)

Agouti-related protein (AgRP); Annexin 2 (ANXA2; a member of a super-family of calcium-dependent membrane binding proteins participating to a variety of fundamental biological process, including cell survival and apoptosis); Autophagosome protein (LC3); Autophagy related-protein (Atg); brown adipose tissue (BAT); high-fat diet (HFD); inducible nitric oxide synthase (INOS; associated with NAFLD); IkappaB kinase beta (IKKβ); intracerebroventricular (i.c.v); knockout (KO); neuropeptide (NP)-Y; NOD-, LRR- and, pyrin domain-containing protein 3 (NLRP3); non-alcoholic fatty liver disease (NAFLD); non-alcoholic steatohepatitis (NASH); pink1 (gene involved in mitophagy); reactive oxygen species (ROS); (+/−) haploinsufficency; ↑ increase, ↓ decrease.

Although the majority of the studies reported in the present review have been performed in laboratory animals it is reasonable to hypothesize their future clinical practice application; this latter concept is strongly supported by the observation that drugs already on the market, used for the control of obesity and other metabolic disorders, are also able to facilitate autophagy processes by dampening inflammation. This evidence strongly contributes to the idea that targeting autophagy and inflammation might represent a druggable system for the management of such diseases.

However, the complex nature of obesity and metabolic disorders might represent a limit of the studies reported here; indeed, although many pharmacological treatments, producing positive metabolic effects, are also able to modulate autophagic flux and inflammation, it is not clear if the final beneficial effect might occur only by their mechanism of action, rather than because of additionally involved pathways. In addition, most of the drugs targeting autophagy and inflammation do not directly modulate such processes but rather affect upstream involved pathways, thus suggesting a more intricate view that deserves further exploration. This concept should foster future studies aimed at a better understanding of whether the beneficial effect accounts because of the selective pharmacological modulation of one of the two processes (autophagy or inflammation) which in turn regulate the other or if they are the result of a concerted action of the same drug on the two systems at the same time.

These studies might help to better identify the molecular mechanism of action of drugs already on the market and, might open the way for the development of novel pharmacological approaches for the treatment of obesity and metabolic diseases.

## Abbreviations

Acid palmitate (PA); Agouti-related protein (AgRP); AMP-activated protein kinase (AMPK); Annexin 2 (ANXA2); Autophagy-related proteins (ATGs); Brown adipose tissue (BAT); Endoplasmic reticulum (ER); Glucagon-like peptide (GLP)-1; GLP-1 receptor (GLP1R); High-fat diet (HFD); IkappaB kinase beta (IKKβ); Inducible NOS (INOS); Interleukin (IL); Intracerebroventricular (i.c.v.); Lipid droplets (LDs); Mammalian target of rapamycin (mTOR); Neuropeptide Y (NPY); Nitric oxide synthase (NOS); Non-alcoholic fatty liver disease (NAFLD); Non-alcoholic steatohepatitis (NASH); NOD-like receptors (NLRs); Oleoylethanolamide (OEA); Peroxisome proliferator-activated receptor alpha (PPAR-alpha); Reactive oxygen species (ROS); Sodium-glucose cotransporter 2 (SGLT2); Toll-like receptors (TLRs); Triacylglycerols (TGs); Triggering receptor expressed on myeloid cells (TREM); Type 2 diabetes mellitus (T2DM); White adipose tissue (WAT).
